# Carbohydrates stimulated Amaryllidaceae alkaloids biosynthesis in *Leucojum aestivum* L. plants cultured in RITA^®^ bioreactor

**DOI:** 10.7717/peerj.8688

**Published:** 2020-03-16

**Authors:** Agata Ptak, Emilia Morańska, Edyta Skrzypek, Marzena Warchoł, Rosella Spina, Dominique Laurain-Mattar, Magdalena Simlat

**Affiliations:** 1 Department of Plant Breeding, Physiology and Seed Science, University of Agriculture in Krakow, Krakow, Poland; 2 The Franciszek Górski Institute of Plant Physiology Polish Academy of Sciences, Krakow, Poland; 3 Université de Lorraine, CNRS, L2CM, Nancy, France

**Keywords:** *Leucojum aestivum*, In vitro, Carbon sources, Galanthamine, Lycorine

## Abstract

**Background:**

*Leucojum aestivum* L. is an important medicinal plant which produces Amaryllidaceae alkaloids, especially galanthamine and lycorine. Research is currently exploring the possibility of producing these alkaloids using biotechnological methods, including in vitro cultures. The biosynthesis of alkaloids may be affected by the types and concentrations of carbohydrate sources used in the medium. In the present investigation we performed such studies on in vitro cultures of *L. aestivum* with a view to obtaining plant material of good quality, characterized, in particular, by a high content of valuable Amaryllidaceae alkaloids.

**Methods:**

We examined the effects of various types of carbohydrate sources—sucrose, glucose, fructose and maltose—at different concentrations (30, 60 and 90 g/L)—on the quality of *L. aestivum* plants grown in the RITA^®^ bioreactor. The plants’ quality was assessed by their biomass increments, as well by as analysing photosynthetic pigments, endogenous sugar, phenolics and Amaryllidaceae alkaloid content. We also investigated the effect of sugars on the activity of the antioxidant enzymes catalase (CAT), peroxidase (POD) and superoxide dismutase (SOD).

**Results:**

The highest biomass increments were observed in plants cultivated in the medium containing 90 g/L sucrose. The highest CAT activity was noted in cultures growing in the medium supplemented with 90 g/L maltose, while the highest POD activity was observed in the presence of 90 g/L fructose and 60 g/L maltose. No differences in SOD activity were observed. Moreover, the sugars did not affect the contents of chlorophyll *a* and carotenoids, whereas the highest amount of chlorophyll *b* was recorded in plants growing in the medium with 60 g/L maltose. No statistically significant differences were observed in the contents of endogenous sugars and phenolics in any in vitro conditions. However, the addition of sugar had a decisive effect on the biosynthesis of the Amaryllidaceae alkaloids. The highest distribution of alkaloids occurred in plants cultured in the medium containing 60 g/L sucrose. Six Amaryllidaceae alkaloids were detected in the plant tissue. The addition of 30 g/L fructose in the medium resulted in the accumulation of five alkaloids, including ismine, which was not identified in other analysed tissues. The highest concentration of galanthamine was observed in plants cultured in the presence of 30 g/L fructose and 60 g/L sucrose (39.2 and 37.5 µg/g of dry weight (DW), respectively). The plants grown in the medium containing 60 g/L sucrose exhibited the highest lycorine content (1048 µg/g of DW).

**Conclusions:**

The type and concentration of sugar used in the medium have an essential influence on the biosynthesis of Amaryllidaceae alkaloids in *L. aestivum* plants cultured in a RITA^®^ bioreactor. The results point to an interesting approach for commercial production of galanthamine and lycorine.

## Introduction

*Leucojum aestivum* L. (summer snowflake), a member of the Amaryllidaceae family, is known for the production of pharmacologically active alkaloids. More than 500 Amaryllidaceae alkaloids have been isolated and some of them displayed bioactivities such as antitumor, antiviral, antibacterial, antifungal, antimalarial and analgesic functions (Laurain-Mattar & Ptak, 2018). However, so far, the most important alkaloids are galanthamine and lycorine. Galanthamine, an acetylcholinesterase inhibitor, is used for the treatment of Alzheimer’s disease (Luttmann, Linnemann & Fels, 2002; Heinrich & Teoh, 2004; Seltzer, 2006). Conversely, lycorine has antiviral and antimalarial properties and may inhibit cell division. Currently, clinical trials are being carried out to determine the use of lycorine in cancer treatment (Ying et al., 2017).

The in vitro culture of *L. aestivum*, as an alternative to chemical synthesis and the extraction of alkaloids from plant materials, has been successfully established (Pavlov et al., 2007; Ptak et al., 2009). The production of secondary metabolites and biomass may be enhanced by in vitro variations as chemical and physical agents. In the studies on biosynthesis of Amaryllidaceae alkaloids in in vitro cultures of *L. aestivum* the influence of mineral nutrients, auxins, cytokinins, ethylene and melatonin was tested (Laurain-Mattar & Ptak, 2018). Elicitation with methyl jasmonate and salicylic acid, and biotransformation using precursor 4′-*O*-methylnorbelladine were also studied (Saliba et al., 2016; Ptak et al., 2017). Additionally, the effects of physical factors, such as temperature and physical state of the medium, on the production of galanthamine and lycorine, in particular, were studied (Laurain-Mattar & Ptak, 2018). Furthermore, successful transformation of *L. aestivum* with *Agrobacterium rhizogenes* was performed (Diop et al., 2007).

Also, sugar—its type and concentration in the medium—is an important factor which may affect the biosynthesis of secondary metabolites. Plant cell cultures are usually grown heterotrophically using simple sugars such as glucose, fructose, maltose, sucrose as an energy and carbon source (Murthy et al., 2014). Sugars also have important functions in the biosynthetic pathways of many compounds by regulating the expression of a significant number of genes (Koch, 1996; Calamar & De Klerk, 2002; Rolland, Moore & Sheen, 2002). In most cases, elevated levels of sucrose enhanced the production of metabolites. For example the accumulation of benzophenanthridine alkaloids (*Eschscholzia californica*), indole alkaloids (*Catharanthus roseus*), rosmarinic acid (*Coleus blumei* and *Eryngium planum*), anthocyanins (*Aralia cordata* and *Hibiscus subdariffa*), ginseng saponins and polysaccharides (*Panax notoginseng*) in the in vitro cultures were enhanced by sucrose at higher concentrations (Zhang, Zhong & Yu, 1996; Kikowska et al., 2012; Bhatia & Bera, 2015). On the other hand, for the adventitious root cultures of *Echinacea angustifolia* high sucrose concentration (70 g/L) was more favourable for biomass accumulation, whereas 50 g/L sucrose was best for the biosynthesis of phenols and flavonoids (Wu et al., 2006). Often, the biosynthesis of secondary metabolites is stimulated by stress conditions that can be observed when high levels of sugar are used in the medium (Khan et al., 2018). The biosynthesis of metabolites may also be affected by carbon sources. In the studies on *Arnica montana* hairy root cultures, the influence of sucrose and glucose was found to be favourable for the production of flavonoids (Wang & Weathers, 2007). The addition of glucose increased the total alkaloid yield in the *Catharanthus roseus* cell culture (Mishra, Srivastava & Akhtar, 2018). The level of artemisinin in the hairy root cultures of *Artemisia annua* was twice when the medium was supplemented with fructose than with sucrose (Wang & Weathers, 2007).

Our earlier research focused on determining the effect of sucrose concentration (30, 60, 90 and 120 g/L) on Amaryllidaceae alkaloid biosynthesis in *L. aestivum, Narcissus pseudonarcissus* and *Galanthus elwesii* cultures grown on solid media (El Tahchy et al., 2011). The addition of 60 g/L of sucrose to the culture medium promoted alkaloid diversity in *G. elwesii* shoot cultures. In that case we identified four Amaryllidaceae alkaloids: galanthamine, trispheridine, crinine and demethylmaritidine. It is also worth emphasizing that in shoots grown on the medium containing 30 g/L of sucrose we did not identify any alkaloids. In addition, enrichment of the medium with 60 and 90 g/L sucrose stimulated galanthamine biosynthesis in *G. elwesii* cultures. In the case of *L. aestivum* shoot cultures the same effect was obtained with the addition of 60 g/L of sucrose. On the other hand, the shoots of *N. pseudonarcissus* produced the highest galanthamine content when they were cultured in the presence of 30 g/L sucrose (El Tahchy et al., 2011).

According to the available literature, there are no reports on the effects of different carbohydrate sources and their concentrations on Amaryllidaceae alkaloid biosynthesis. In view of an increase in the scale of alkaloid production, it is advisable to use a bioreactor for such research (Georgiev et al., 2012). Liquid media are ideal for micropropagation as they affect a reduction in the costs of both biomass and secondary plant metabolite production (Georgiev et al., 2012; Murthy et al., 2014). To date, studies have been conducted only on the effect of sucrose concentration in shaken shoot cultures of *N. confusus* and *L. aestivum* (Sellés et al., 1997; Schumann et al., 2012). Studies on the adaptation of the temporary immersion system, RITA^®^, for growing *L. aestivum* shoot and plant cultures were carried out by Ivanov et al. (2011) and Ptak et al. (2017). However, the results of the research conducted by Ivanov et al. (2012) and Georgiev et al. (2014) on shoot cultures of *L. aestivum*, as well as *Pancratium maritimum*, with the use of standard amount of sucrose in the medium, showed that the optimal conditions for growth in the RITA^®^ bioreactor (immersion frequency and stand-by periods) provided both the optimal biomass accumulation and the best sugar utilization from the medium. Vishnevetsky, Zamski & Ziv (2003) reported that in the case of *Nerine sarniensis* bulbs developing in a liquid culture, sucrose in the medium was hydrolyzed to glucose and fructose and total soluble sugars after 8 weeks decreased from 60 g/L to approximately 39 g/L. On the other hand, according to Ziv (2005), sucrose is reduced or rapidly removed from the medium in both agar-gelled and liquid cultures. Georgiev et al. (2014) also suggest that in *P. maritimum* shoot cultures grown in the RITA^®^ bioreactor sugar metabolism is based not only on the consumption of sugars from the culture medium but also on self- biosynthesis of sugars resulting from the running photosynthesis.

The very nature of liquid tissue culture is an imposing stress to which the plants respond to the environmental signals in developmental aberration. The submerged tissue was found to exhibit oxidative stress symptoms, with elevated levels of reactive oxygen species that were associated with a change in antioxidant enzyme activity (Ziv, 2005). According to the literature data, extracellular sugars can influence the metabolism of the cultures through changes in the osmotic environment of biological systems (Georgiev et al., 2014). It is known that osmotic stress produced by sucrose alone in combination with other stress factors, such as liquid culture, can affect the biosynthesis of secondary metabolites (Naik & Al-Khayri, 2016; Isah et al., 2018).

Our study for the first time demonstrated the influence of various types of carbohydrates (sucrose, glucose, fructose and maltose) and their concentrations (30, 60 and 90 g/L) on the biomass increments, antioxidant enzymes: catalase (CAT), peroxidase (POD) and superoxide dismutase (SOD) activities, endogenous sugars content, chlorophyll *a*, chlorophyll *b*, carotenoids, phenolics and Amaryllidaceae alkaloids biosynthesis in *L. aestivum* plants cultured in bioreactor RITA^®^.

## Materials and Methods

### Induction of plant cultures

Somatic embryogenesis was induced on leaf explants isolated from *L. aestivum* bulbs. All the stages of somatic embryogenesis (induction and multiplication of embryogenic callus, induction of somatic embryos and their conversion into plants) were carried out according to the procedure described previously by Ptak et al. (2013). The obtained plants were grown on Murashige & Skoog (1962) solid medium containing 5 µM of zeatin for 12 months. The plants were transferred onto fresh medium every 4 week. After that time they were used for carrying out the experiment.

### Treatment and culture conditions

Twelve-month-old plants were transferred to liquid medium, its composition being the same as that of the solid medium, used at the stage of the plant growth, but enriched with different carbohydrates: sucrose, glucose, fructose or maltose at concentrations of 30, 60 or 90 g/L.

Experiment was carried out in RITA^®^ bioreactor with temporary immersion system (Vitropic, France). For each a RITA^®^ vessels 200 mL of medium and 5 g of plants were used. The physical parameters used for the culture were previously described (Ptak et al., 2017). The experiment was established in three replications. After 1 month of culture, the fresh weight (FW) increments of plants were determined according to the formula: final fresh weight minus initial fresh weight. The samples from each treatment were taken for biochemical and phytochemical analyses. Each analyses was repeated three times.

### Biochemical analyses

#### Determination of sugars

Plant tissue (200 mg) was extracted with 2 mL of 80% aqueous ethanol, and then it was centrifuged at 2,800 rpm for 10 min. The amounts of total soluble sugars were estimated by the phenol-sulphuric method (Dubois et al., 1956). The absorbance (λ = 490 nm) of the samples was measured spectrophotometrically (Evolution 300; Thermo Fisher Scientific, Waltham, MA, USA). The amounts of soluble sugars were determined against a glucose standard curve and expressed in milligrams per gram of FW.

#### Analyses of antioxidant enzymes: CAT, POD, SOD

For the analyses of enzymes 100 mg of fresh plant tissue was homogenized at 4 °C with phosphate buffer (pH 7.8) containing 0.01 M EDTA and 0.5% BSA. The homogenate was centrifuged at 10,000 rpm for 15 min. Activity of CAT, POD and SOD was measured spectrophotometrically (the absorbances were: λ = 240, 485 and 595 nm, respectively) (Lück, 1962; McCord & Fiodovich, 1969; Aebi, 1984). One unit was defined as the amount of enzyme necessary for 50% inhibition of cytochrome *c* in a coupled system with xanthine and xanthine oxidase. The reaction kinetics for all the enzymes was examined 60 s after initiation of the reaction.

#### Determination of pigments: chlorophylls a, b and carotenoids

Chlorophylls and carotenoids were extracted from 100 mg of fresh tissue samples in 1 mL of 80% ethanol for 12 h. After centrifugation (15,000 rpm and 15 min), an aliquot of the extract was added to microplate wells and absorbance was measured spectrophotometrically at 470, 648 and 664 nm on a microplate reader (Synergy 2, Bio-Tek, Winooski, VT, USA). The concentrations of chlorophylls *a, b* and carotenoids were determined according to the method of Lichtenthaler & Wellburn (1983) and expressed per gram of FW.

#### Determination of total phenolic compounds

Two hundred mg of plant tissue was homogenized in 2.0 mL of 80% ethanol and centrifuged at 2,800 rpm for 20 min. The supernatant was mixed with 20% Na_2_CO_3_ and Folin-Ciocalteu reagent (Singleton & Rossi, 1965). The absorbance (λ = 760 nm) of the samples was estimated spectrophotometrically (Evolution 300; Thermo Fisher Scientific, Waltham, MA, USA) according to Singleton & Rossi (1965) with modifications. The total phenolic content was calculated as milligrams of chlorogenic acid per gram of FW.

### Phytochemical analyses

The alkaloids were extracted from plants as previously described by El Tahchy et al. (2011). The total methanol extracts were used for SPE purification (Solid Phase Extraction). The purified methanol extracts were analyzed using a gas chromatography–mass spectrometry (GC–MS) system according to Saliba et al.’s (2016) method. The alkaloids were identified by comparing the measured data with those of authentic compounds (galanthamine and lycorine) and with the help of the NIST08.LIB database (Ptak et al., 2009, 2019).

### Data analysis

The results are expressed as mean values and standard deviation (SD). The values were subjected to analysis of variance (ANOVA). Differences between the means were performed using Duncan’s multiple range test at *p* < 0.05.

## Results

**Figure 1:**
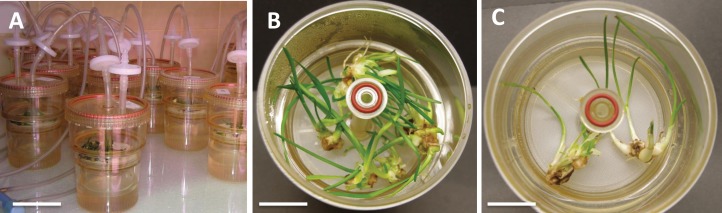
*L. aestivum* plants after 4 weeks of cultivation in bioreactor RITA^®^. (A) RITA^®^ vessels, bar = 3 cm, (B) plants growth in medium with 90 g/L sucrose, bar = 1 cm, (C) plants growth in medium with 90 g/L maltose, bar = 1.5 cm.

**Figure 2:**
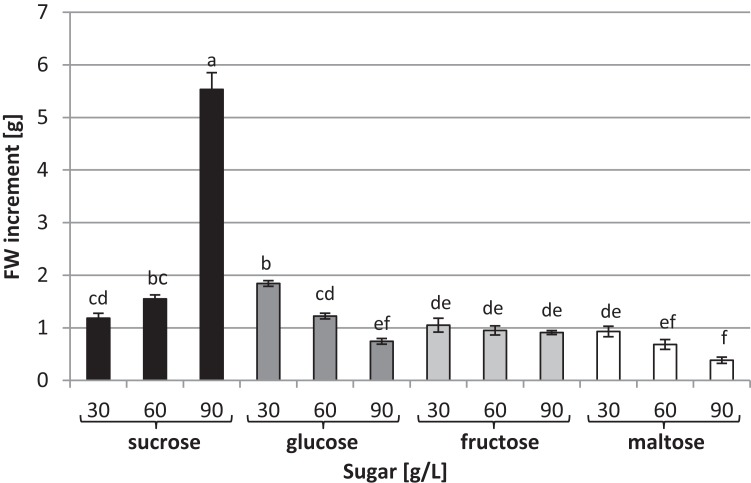
Effects of sugar type and their concentration on fresh biomass increments of *L. aestivum* in vitro plants. The results are means of three replicates (*n* = 3). Error bars represent ± SD. Different letters indicate a significance difference at *p* < 0.05 according to ANOVA and Duncan’s test.

To investigate the effects of various carbon sources at different concentrations on in vitro cultures of *L. aestivum*, sucrose, glucose, fructose and maltose at concentrations of 30, 60 and 90 g/L were added to a RITA^®^ bioreactor (Fig. 1A). The highest fresh biomass increments (5.53 g) were observed in plants cultured in the presence of 90 g/L sucrose (Figs. 1B and 2), while the lowest (0.38 g) were detected in the plants cultured in the medium enriched with 90 g/L maltose (Figs. 1C and 2). At the conventional concentration of sucrose in the medium (30 g/L), the biomass increments were 1.18 g (Fig. 2).

**Figure 3:**
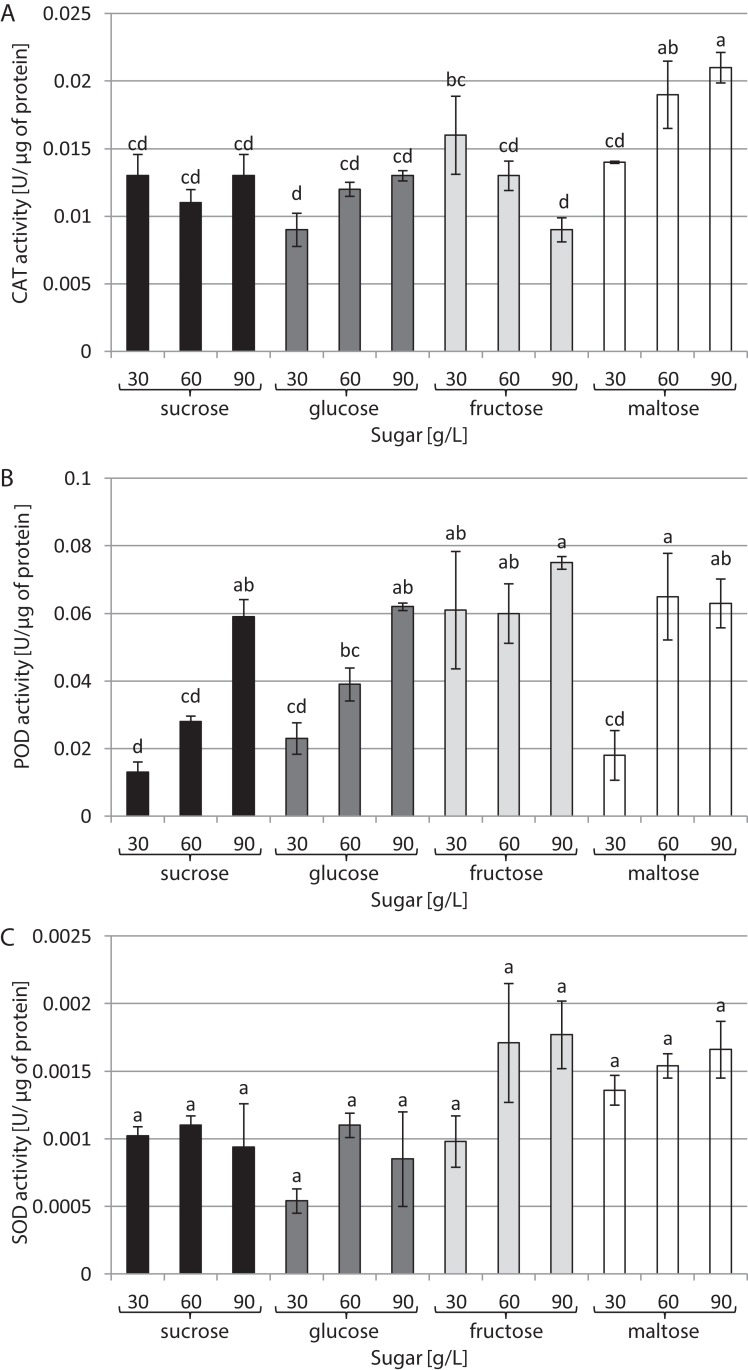
Effects of sugar type and their concentration on antioxidant enzyme activities. (A) CAT, (B) POD, (C) SOD. The results are means of three replicates (*n* = 3). Error bars represent ± SD. Different letters indicate a significance difference at *p* < 0.05 according to ANOVA and Duncan’s test.

The type of sugar used in the medium and its concentration had a decisive impact on CAT activity (Fig. 3A). The highest activity (0.021 U/µg of protein) was noted in cultures growing in a medium supplemented with 90 g/L maltose. In contrast, the lowest CAT activity (0.009 U/µg of protein) was found in cultures growing in media supplemented with 30 g/L glucose and 90 g/L fructose. The highest activity of POD was observed in the presence of 90 g/L fructose and 60 g/L maltose: 0.075 and 0.065 U/µg of protein, respectively. The plants grown in a medium with 30 g/L sucrose exhibited the lowest POD activity (0.003 U/µg of protein) (Fig. 3B). The activity of SOD was at the same level in all tested conditions, amounting to 0.001 U/µg of protein on average (Fig. 3C).

**Figure 4:**
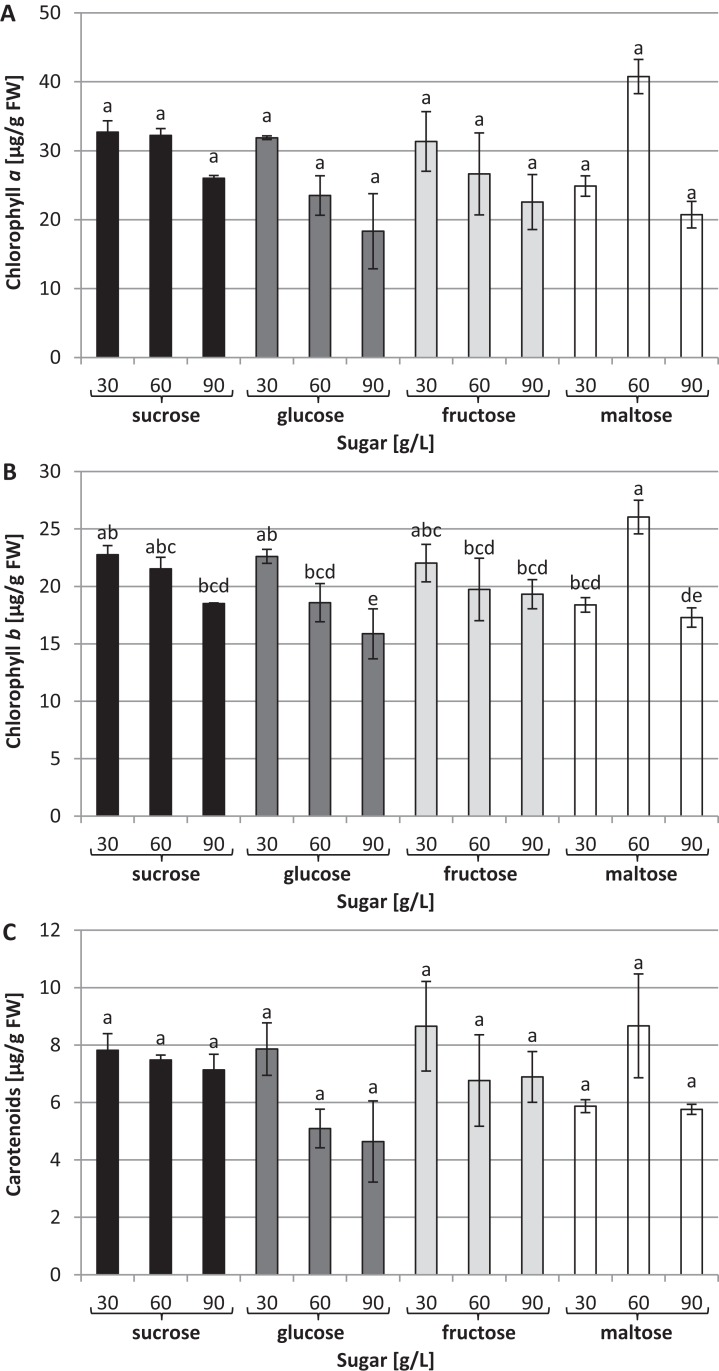
Effects of sugar type and their concentration on pigments content. (A) chlorophyll *a*, (B) chlorophyll *b*, (C) carotenoids. The results are means of three replicates (*n* = 3). Error bars represent ± SD. Different letters indicate a significance difference at *p* < 0.05 according to ANOVA and Duncan’s test.

The chlorophyll *a* concentration remained at the same average level: approximately 27.63 µg/g of FW (Fig. 4A). On the other hand, interrelationships between carbon sources, their concentrations and chlorophyll *b* content were observed (Fig. 4B). The highest chlorophyll *b* content (26.04 µg/g of FW) was recorded in plants grown in a medium enriched with 60 g/L maltose, while the lowest concentration (15.87 µg/g of FW) was observed in plants cultured in a medium containing 90 g/L glucose. The carotenoid content was the same irrespective of the type and concentration of sugar: approximately 6.4 µg/g of FW on average (Fig. 4C).

No statistically significant differences were observed in the content of endogenous sugars in the *L. aestivum* plants in any in vitro conditions (Table 1). However, we observed that with the addition of the lowest concentrations of sugar into the medium (30 g/L), endogenous sugars ranged from 119.35 to 122.49 µg/g of FW, while with added sugar concentrations of 90 g/L, the contents ranged between 127.27 and 133.98 µg/g of FW. The type of carbon sources used in the medium had no effect on the content of total soluble sugars in the plants.

**Table 1:** Effects of sugar types and their concentrations on total soluble sugar and phenolics content in *L. aestivum* plants.

Type of sugar	Concentration (g/L)	Total soluble sugars (µg/g FW)	Total phenolics (µg/g FW)
Sucrose	30	120.95 ± 1.98a*	7.13 ± 0.08a
	60	128.56 ± 3.73a	7.29 ± 0.07a
	90	132.38 ± 1.36a	6.82 ± 0.11a
Glucose	30	122.49 ± 1.90a	7.27 ± 0.14a
	60	133.90 ± 3.35a	7.11 ± 0.10a
	90	132.58 ± 5.70a	7.26 ± 0.16a
Fructose	30	119.34 ± 2.05a	7.15 ± 0.12a
	60	130.41 ± 4.59a	7.31 ± 0.21a
	90	133.99 ± 1.93a	7.61 ± 0.14a
Maltose	30	120.65 ± 2.20a	7.39 ± 0.06a
	60	125.81 ± 1.12a	7.29 ± 0.18a
	90	127.27 ± 0.74a	7.25 ± 0.18a

**Note:**

* The results are means of three replicates (*n* = 3) ± SD. Different letters indicate a significance difference at *p* < 0.05 according to ANOVA and Duncan’s test.

None of the tested conditions affected the phenolic compounds biosynthesis in the plants either. The average accumulation in all cultures was approximately 7.24 µg/g of FW (Table 1).

Using capillary GC–MS, we identified seven alkaloids in the obtained plant materials: galanthamine, lycorine, tazettine, ismine, crinine, dimethylmaritidine and haemanthamine (Table 2). The identification was performed by comparing the measured data with the help of the NIST08.LIB database and with data from the literature. Plants grown in the medium containing 60 g/L sucrose showed the highest distribution (Table 2), with six Amaryllidaceae alkaloids identified in their tissues. In plants grown in the medium with 30 g/L fructose, five alkaloids were found. It is noteworthy that ismine was only detected in those plants. The fewest alkaloids were found in plant material treated with 30 and 60 g/L glucose and 30 and 90 g/L maltose.

**Table 2:** Alkaloids identified in *L. aestivum* plant cultures.

Alkaloids	Formula	Base peaks	Treatments											
			1*	2	3	4	5	6	7	8	9	10	11	12
Galanthamine	C_17_H_21_NO_3_	286.00	+	+	+	+	+	+	+	+	+	+	+	+
Lycorine	C_16_H_17_NO_4_	226.00	+	+	+	+	+	+	+	+	+	+	+	+
Tazettine	C_18_H_21_NO_5_	247.00	+	+	−	−	−	+	+	+	+	−	+	−
Ismine	C_15_H_15_NO_3_	238.10	−	−	−	−	−	−	+	−	−	−	−	−
Crinine	C_16_H_17_NO_3_	286.00	−	+	−	−	−	−	−	−	−	−	−	−
Dimethylmaritidine	C_16_H_19_NO_3_	272.90	−	+	−	−	−	−	+	−	−	−	−	−
Haemanthamine	C_17_H_19_NO_4_	124.05	−	+	−	−	−	−	−	−	−	−	−	−

**Note:**

* 1: sucrose 30 g/L, 2: sucrose 60 g/L, 3: sucrose 90 g/L, 4: glucose 30 g/L, 5: glucose 60 g/L, 6: glucose 90 g/L, 7: fructose 30 g/L, 8: fructose 60 g/L, 9: fructose 90 g/L, 10: maltose 30 g/L, 11: maltose 60 g/L, 12: maltose 90 g/L.

**Figure 5:**
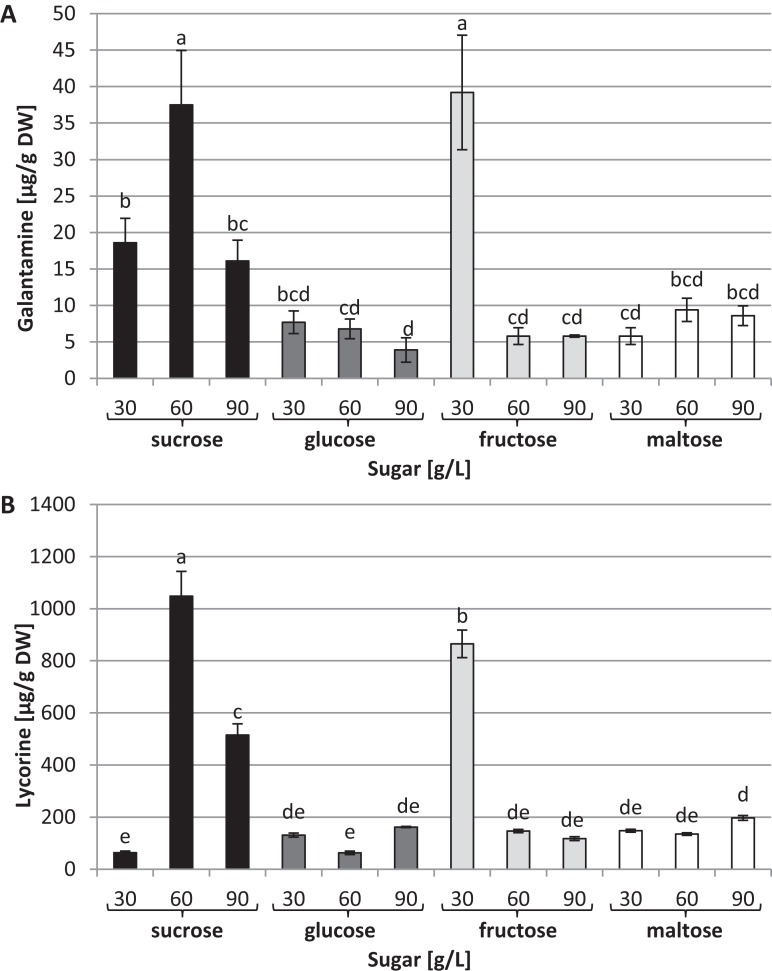
Effects of sugar type and their concentration on alkaloids content. (A) Galanthamine, (B) lycorine. The results are means of three replicates (*n* = 3). Error bars represent ± SD. Different letters indicate a significance difference at *p* < 0.05 according to ANOVA and Duncan’s test.

Capillary GC–MS was also used to identify and quantify galanthamine and lycorine in tissue cultures through a comparison with authentic compounds. These two alkaloids were detected in all the conditions tested; however, their concentrations varied dramatically depending on the type and concentration of the carbon source used in the medium (Fig. 5). The highest levels of galanthamine were detected in plants grown in the media with 30 g/L fructose and 60 g/L of sucrose (39.2 and 37.5 µg/g of DW, respectively). These contents were about 9.8 times higher than those observed in plants grown in the medium with 90 g/L glucose (Fig. 5A). The highest lycorine content was found in plants grown in the medium containing 60 g/L sucrose (1,048 µg/g of DW). This amount was approximately 16.53 times higher than the content found in plants grown in the medium with 30 g/L sucrose (Fig. 5B).

## Discussion

This study examined the effects of various types and concentrations of sugar on *L. aestivum* plants grown in a RITA^®^ bioreactor. The maximum biomass was recorded in plants grown in a medium containing 90 g/L sucrose. Sucrose is the most popular carbon source used in plant tissue cultures. In many studies on in vitro cultures of such plants as *Dendrobium huoshanense*, *Withania somnifera*, *Solanum elaeagnifolium* and *Oryza sativa*, sucrose was found to be the best source of carbon (Nigra, Alvarez & Giulietti, 1990; Jain et al., 1997; Zha et al., 2007; Nagella & Murthy, 2010). Varshney et al. (2002) found that for the multiplication of *Lilium* bulblets, sucrose was more effective than glucose or fructose, and that the concentration was an important factor; whereas usually a concentration of 30 g/L is used, in *Lilium* cultures, depending on the cultivar, 60–90 g/L sucrose was found to be optimum. High concentrations of sucrose are also necessary for bulb formation in *Narcissus* (Chow, Selby & Harvey, 1992; Sellés et al., 1997), *Tulipa* (Bach & Ptak, 2005) and *Leucojum vernum* (Ptak, 2010) plants grown in vitro. A concentration of 50 g/L sucrose is beneficial for *Fagonia indica* callus growth (Khan et al., 2018). However, sucrose at high concentrations can be toxic and inhibit the growth and development of plants. This phenomenon was observed, for example in *Metroxylon sagu* plants grown in vitro (Sumaryono, Muslihatin & Ratnadewi, 2012). In our experiment, maltose at a concentration of 90 g/L clearly reduced the *L. aestivum* plants’ biomass. On the other hand, maltose at higher concentrations had a positive effect on *F. indica* and *Gossypium hirsutum* callus growth (Kumar et al., 2015; Khan et al., 2018).

Apart from their important role in in vitro cultures as sources of energy and carbon, sugars also act as osmotic agents and may induce osmotic stress when they are used at high concentrations in the medium (Baque et al., 2012). Plants have an effective enzymatic antioxidant defence system involving CAT, POD and SOD. This system allows scavenging of reactive oxygen species (ROS), protecting plant cells from oxidative damage (Bolouri-Moghaddam et al., 2010). In this study, the CAT activity of *L. aestivum* plants was optimally enhanced by 90 g/L maltose compared to other carbohydrate sources and concentrations, while the highest POD activity was observed under the influence of 60 g/L maltose and 90 g/L fructose. It is, however, worth noting that the high activity of CAT and POD was accompanied by a decrease in biomass, which may indicate potential negative effects of stress when high concentrations of maltose and fructose were used in the medium. On the other hands no significant change in SOD activity was observed with any of the sugar types and concentrations used.

The types and concentrations of sugar affected the content of chlorophyll *b* in the *L. aestivum* plants. The highest content was observed in plants grown in the medium with 60 g/L maltose, while 90 g/L glucose inhibited the biosynthesis of chlorophyll *b*. This is in line with Khan et al. (2018), who found that high glucose levels in the medium inhibited the biosynthesis of chlorophyll *b* in callus cultures of *F. indica*. Other studies reported that the concentration of sugar can affect the synthesis of chlorophyll. A high sucrose concentration in the medium impairs the photosynthetic efficiency of cultured plants by reducing the levels of chlorophyll and promoting the formation of abnormal stomata (Hazarika, 2006). On the other hand, the leaves of *Pogostemon cablin* plants cultured in a medium enriched with glucose and fructose had lower total chlorophyll content compared with sucrose, regardless of the concentration used (Swamy et al., 2010). In contrast, in our study, the concentration of chlorophyll *a* and carotenoids remained at the same level in all cultures tested. In a study on callus cultures of *Calendula officinalis*, carotenoid production gradually increased with a progressive increase in the sucrose concentration (Legha et al., 2012). In contrast, in our study none of the sucrose concentrations used in the medium affected the carotenoid biosynthesis of *L. aestivum* plants.

The types and concentrations of sugars used in the medium did not significantly affect the soluble carbohydrate content in the *L. aestivum* plants. Soluble carbohydrates are synthesised in response to osmotic stress, protecting cellular members and improving survival under stress conditions (Valluru & Van den Ende, 2008). We observed that higher concentrations of sugar in the medium increased the content of endogenous sugar in the plants, although the relationship was statistically insignificant. In shoot cultures of *Dendrobium* and *Billbergia zebrina*, the soluble carbohydrate content increased concomitantly with higher sucrose concentrations in the medium (Ferreira et al., 2011; Martins et al., 2015). However, the total soluble carbohydrate content varied during the induction and development of protocorm-like bodies of *Cattleya tigrina* cultured in vitro. A decline of the soluble sugar concentration was observed only on the 100th day of culture (De Conti et al., 2018).

It has long been established that sugars are involved in the expression of genes implicated in both primary and specialised metabolisms (Ali et al., 2016). In our research, the different types and concentrations of carbohydrates did not affect the total phenolics content in *L. aestivum* plants. Conversely, the maximum phenolic compounds content in suspension cultures of *Artemisia absinthium* was observed in the presence of 50 and 70 g/L sucrose and maltose, respectively (Ali et al., 2016). The high sucrose concentration also enhanced the accumulation of phenolic compounds in the adventitious roots of *Echinacea angustifolia* cultures (Wu et al., 2006).

The highest distribution of Amaryllidaceae alkaloids was recorded in the *L. aestivum* plants cultivated in the medium with 60 g/L sucrose. Six alkaloids were identified in these cultures. A stimulating effect of 30 g/L fructose on alkaloid biosynthesis was also recorded: five alkaloids were identified, including ismine, which was not present under any other conditions.

This is the first study to explore the effects of different types and concentrations of sugar on Amaryllidaceae alkaloid biosynthesis in cultures of *L. aestivum* grown in a RITA^®^ bioreactor. Our earlier studies concerned the effect of different sucrose concentrations on the biosynthesis of alkaloids in *L. aestivum* shoots grown in solid media, reporting the highest in vitro diversity—with four alkaloids identified—in shoots treated with 30 g/L sucrose (El Tahchy et al., 2011). It is worth emphasizing that in our previous studies on *L. aestivum* shoot cultures grown in solid media with 90 g/L sucrose, we did not identify galanthamine or lycorine. The fact that these alkaloids were identified in plants grown in the RITA^®^ bioreactor with the same concentrations of sucrose confirms our previous results suggesting that not only the type and concentration of the elicitor used, but also the physical condition of the medium has a decisive impact on the biosynthesis of Amaryllidaceae alkaloids (Ptak et al., 2013). In addition, the use of bioreactor cultures in combination with 60 g/L sucrose or 30 g/L fructose resulted in the synthesis of alkaloids that we did not observe in our previous experiment with any sucrose concentration.

In this study, the highest galanthamine content in *L. aestivum* plant cultures was found in media with 30 g/L fructose and 60 g/L sucrose. Galanthamine biosynthesis was also stimulated by 60 g/L of sucrose in *L. aestivum* shoot cultures grown in a solid medium (El Tahchy et al., 2011). Studies on the effect of sucrose concentrations on galanthamine biosynthesis in agitated shoot cultures of *N. confusus* found the highest level of this alkaloid in tissue grown in a medium containing 90 g/L sucrose (Sellés et al., 1997). The concentration of sucrose in the medium was also of importance for terpenes biosynthesis in *Ginkgo biloba* cell cultures (Park et al., 2004). The effect of fructose on galantamine biosynthesis has not previously been studied. Fructose at a concentration of 30 g/L increased the level of artemisinin in hairy root cultures of *Artemisia annua* (Wang & Weathers, 2007).

In our research, the addition of 60 g/L sucrose to the medium improved lycorine production in *L. aestivum* plants. No previous study has examined the effect of sugars on lycorine biosynthesis in Amaryllidaceae plants.

## Conclusions

The addition of sugars to culture media are one of the factors that directly affect the quality of *L. aestivum* growth in a RITA^®^ temporary immersion system, particularly with respect to the plant’s biosynthetic ability. Adding 60 g/L sucrose to the medium in bioreactor RITA^®^ offers new prospects for improving the biosynthesis of Amaryllidaceae alkaloids. In addition, the use of 30 g/L fructose offers the possibility to obtain ismine, which has not been identified in plants grown in media containing other sugar types.

In the future, an integrated approach to process optimization should be adopted using different carbohydrates and their concentrations in the medium with the application of different bioreactor systems for large-scale cultivation of *L. aestivum* plants.

## Supplemental Information

10.7717/peerj.8688/supp-1 Supplemental Information 1 Raw data.

## Additional Information and Declarations

### Data Availability

The following information was supplied regarding data availability: The raw measurements are available in the Supplemental File.
